# Targeting hypoxic response for cancer therapy

**DOI:** 10.18632/oncotarget.7229

**Published:** 2016-02-07

**Authors:** Elisa Paolicchi, Federica Gemignani, Marija Krstic-Demonacos, Shoukat Dedhar, Luciano Mutti, Stefano Landi

**Affiliations:** ^1^ Genetics-Department of Biology, University of Pisa, Pisa, Italy; ^2^ School of Environment and Life Sciences, College of Science and Technology, Cockcroft Building, University of Salford, Salford, UK; ^3^ Department of Integrative Oncology, BC Cancer Research Centre, BC Cancer Agency and Department of Biochemistry and Molecular Biology, Life Sciences Institute, University of British Columbia, Vancouver, British Columbia, Canada

**Keywords:** hypoxia, Warburg effect, cancer stem cells, epithelial mesenchymal transition, clinical trials

## Abstract

Hypoxic tumor microenvironment (HTM) is considered to promote metabolic changes, oncogene activation and epithelial mesenchymal transition, and resistance to chemo- and radio-therapy, all of which are hallmarks of aggressive tumor behavior. Cancer cells within the HTM acquire phenotypic properties that allow them to overcome the lack of energy and nutrients supply within this niche. These phenotypic properties include activation of genes regulating glycolysis, glucose transport, acidosis regulators, angiogenesis, all of which are orchestrated through the activation of the transcription factor, HIF1A, which is an independent marker of poor prognosis. Moreover, during the adaptation to a HTM cancer cells undergo deep changes in mitochondrial functions such as “Warburg effect” and the “reverse Warburg effect”.

This review aims to provide an overview of the characteristics of the HTM, with particular focus on novel therapeutic strategies currently in clinical trials, targeting the adaptive response to hypoxia of cancer cells.

## HYPOXIA AND THERAPEUTIC TARGETS

### Anti-angiogenesis agents and cancer therapy

During the growth of a solid neoplasia, the tumor microenvironment (TM) undergoes biochemical changes that include depletion of glucose, bicarbonate, O_2_ (i.e. hypoxia and anoxia), high levels of lactate and adenosine and low pH values [1, 2]. As a response to hypoxia and nutrient deficiencies, cells activate angiogenesis, that is the development of new blood vessels sprouting from the existing ones [3-6]. However, neo-angiogenesis yields immature vessels with functional and structural alterations, leading to an irregular blood flow, which fails to provide enough oxygen to each cancer cell and the TM remains, essentially, hypoxic [7, 8]. It has been thought that blocking the oxygen supply could be a valid therapeutic strategy to stop cancer progression. Thus, scientific research on cancer treatment has made significant progresses in the development of new anti-angiogenic agents that have entered into clinical practice alone or in combination with the standard chemo-/radio- therapy [9]. Anti-VEGF compounds were developed, such as bevacizumab and ramucirumab as well as the VEGF antagonist aflibercept [7]. However, despite some promising results in clinical trials, the blockade of VEGF signaling yield positive results only in a fraction of solid tumors, such as colorectal, lung and breast HER2+/ER+/PR+ cancers [10, 11]. Moreover, compared to the previous standard of care, treatments based on angiogenesis inhibitors provide poor benefits in terms of progression-free (PFS) and overall survival (OS) [12]. VEGF blockade could exacerbate the oxygen deprivation in the TM leading to a further increased hypoxic state. In these conditions, *in vivo* studies have shown that tumor blood vessel pruning may even stimulate cancer cells to acquire pro-invasive and metastatic traits, a threatening form of tumor adaptation to a hypoxic TM (HTM) [7, 8, 12, 13]. As a matter of fact, it has been shown that glioblastoma patients treated with bevacizumab had a shorter OS [14].

### Hypoxic tumor microenvironment, cancer progression and epithelial-mesenchymal transition

**Figure 1:**
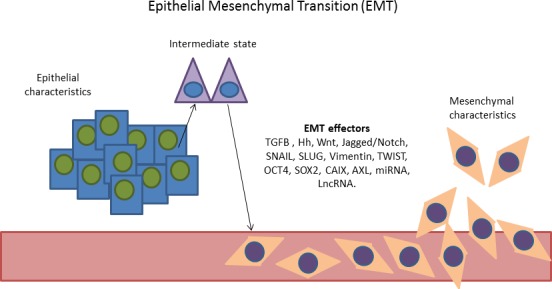
Representation of the epithelial mesenchymal transition Cells with epithelial characteristics acquire mesenchymal characteristics through the activation of EMT effectors. [EMT effectors: TGFB, Hedgehog (Hh), Wnt, Jagged/Notch signalling pathways, SNAIL, Slug, Vimentin, TWIST, OCT4, SOX2, CAIX, AXL, MicroRNAs (miRNA) and long non-coding RNAs (lncRNA)].

**Figure 2:**
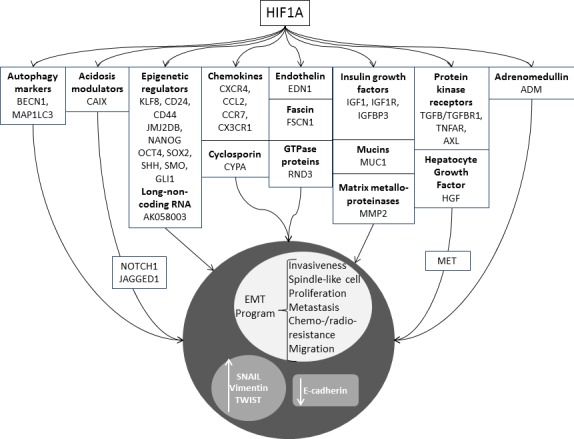
Genes whose expression was shown to be correlated to the activation status of HIF1A, leading to EMT Autophagy markers: *BECN1*, *MAP1LC3*; acidosis modulators: CAIX; epigenetic regulators: KLF8, cell surface glycoproteins (CD24, CD44), lysine (K)-specific demethylase jumonji domain (JMJ2DB), nanog homeobox (NANOG), octamer-binding transcription factor 4 (OCT4), SRY sex determining region Y-box 2 (SOX2), sonic hedgehog (SHH), smoothened, frizzled class receptor (SMO), GLI family zinc finger 1 (GLI1); long non-coding RNA: AK058003; chemokines: CXCR4, CCL2, CCR7, CX3CR1; cyclospporins: cyclophilin A (CYPA); endothelins: endothelin1 (EDN1); fascins: fascin actin-bundling protein 1 (FSCN1); GTPase proteins: Rho family GTPase 3 (RND3); insulin growth factors: IGF1, IGF1R, IGFBP3; mucins: mucin 1, cell surface associated (MUC1); matrix metalloproteinase: MMP2, protein kinases receptors: TGFB/TGFBR1, TNFAR, AXL; HGF; adrenomedullin (ADM).

**Figure 3:**
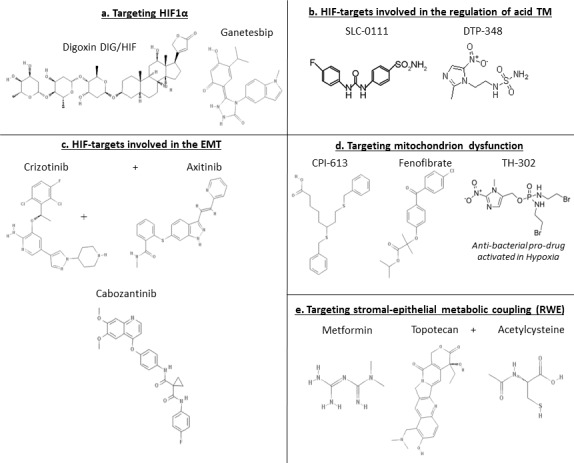
Chemical structures related to Table 1

Following the observations reported above, it was made clear that one of the main problems in fighting cancer is the extreme adaptation of malignant cells to HTM, which promotes malignant progression, and confers increased resistance to chemo- and radio- therapies [15]. In fact, increased hypoxia was correlated to a high expression of markers of epithelial mesenchymal transition (EMT) [16-18] and hypoxic niches were shown to be enriched of cancer stem cells (CSCs) [19, 20]. In summary, cancer cells within a HTM undergo to dramatic changes, ultimately leading to a worsening of their malignant behavior. Thus, nowadays the research is devoted towards the identification of mechanisms involved in the adaptation of cancer cells to the HTM with the aim to detect targets for novel therapeutic agents. A key-molecule identified in mid-nineties is the HIF1A [21]. HIF1A is responsible for the formation of HIF complex that acts as a transcription factor in the activation of a broad spectrum of genes orchestrating large phenotypic changes and ultimately leading to EMT. Following EMT, cells lose their normal phenotype and acquire mesenchymal features [22, 23] including the acquisition of stemness characteristics [24], enhancement of invasiveness and metastasizing capabilities [25]. All these changes are associated with poor prognosis and chemo-/radio-resistance in different tumor models [26, 27]. EMT is characterized by loss of proteins involved in cell adhesion (e.g. E-cadherin) and the over-expression of mesenchymal-specific proteins such as SNAIL, the Vimentin, and TWIST [16, 18]. Phenotypically, these changes correspond to the acquisition of a spindle-like cell morphology, increased invasiveness, migration, and cell proliferation enabling cancer cells to invade surrounding tissues [16]. For this reason, this transition has been regarded as the possible first step in the complex process of generating distant metastases [28, 29] (Figure 1). In parallel with these considerations, it is not surprising that *HIF1A* over-expression was showed to be an independent predictor of poor prognosis in most of the solid tumors [30-34]. The hypoxia>HIF>EMT axis has been studied thoroughly in aggressive tumors [e.g. lung, triple negative breast (TNBC), gastric, ovarian cancer, melanoma, multiple myeloma, malignant pleural mesothelioma (MPM), pancreatic ductal adenocarcinoma (PDAC), hepatocellular and renal cell carcinoma (RCC)] and various important genes activated by HIF complex were identified (Figure 2). Among them, there are. Autophagy markers BECN1 and MAP1LC3 which are activated in lung cancer [35] and in PDAC [36]; the acidosis modulator CAIX, which is overexpressed in TNBC [37] and in RCC [38]; the epigenetic regulators [39] *KLF8, CD24, JMJ2DB* and the long-non coding RNA AK058003 which are overexpressed in gastric cancer [40-43], *CD44* in TNBC [44], *NANOG, OCT4, SRY, SOX2, SHH, SMO, GLI1* in PDAC [45, 46] and MPM [47]; the chemokines *CXCR4* and *CCL2 which* are over-expressed in gastric cancer [48, 49] and multiple myeloma [50], *CCR7* in ovarian cancer [51] whereas *CX3CR1* in PDAC [52]; the cyclosporin *CYPA which* is over-expressed in PDAC [53]; the endothelin *EDN1 which* is activated in melanoma [54]; the fascin *FSCN1 which* is activated in PDAC [55]; the GTPase protein *RND3 which* is over-expressed in gastric cancer [56]; the growth factors related to insulin homeostasis *IGF1*, *IGF1R*, *IGFBP3 which* are over-expressed in lung cancer [57] and hepatocellular carcinoma [58]; the mucin *MUC1 which* is overexpressed in MPM [59], in PDAC [60], in RCC [61] and in lung cancer [62]; the matrix metalloproteinase *MMP2 which* is over-expressed in PDAC, lung cancer and ovarian cancer cell lines [63]; the protein kinase receptors *TGFB/TGFBR1* which are up-regulated in gastric cancer [64], *TNFAR* in melanoma [65] and in RCC [66] and *AXL* in gastric cancer [67], TNBC [68], in lung cancer [69], and MPM [70]. Moreover, another important cell signaling is activated by HIF. It was shown that *HGF*, together with its ligand *MET* (HGF/MET), promotes cell proliferation, survival, migration, and angiogenesis in hepatocellular carcinoma [71]. Furthermore, *ADM* is overexpressed in RCC [72] and in PDAC [73]). *ILK* is activated by *HIF1A* and through a regulatory loop it is responsible for the increase of *HIF1A* expression, in a positive feed-back mechanism [74]. In addition, E-cadherin, which has always been defined as a tumor suppressor, showed an unexpected role in the regulation of genes involved in the response to hypoxia, suggesting a possible novel function in aggressive breast cancer [75]. These recent findings, taken together, prompted clinical researchers to focus on the identification of new compounds able to inhibit *HIF1A* or its targets. Hereon, we will focus on novel therapeutic agents developed for these purposes and for which the clinical trials are in an advanced stage (Table 1, Figure 3). These new molecules could open the way to effective therapies and increase the survival of patients suffering from these aggressive types of cancer.

**Table 1:** Summary of novel therapeutic approaches

Drugs	Targets	Phase	Type of cancer	Ref. study
**a. Targeting HIF1A**				
*Digoxin/DIG-HIF*	HIF1A	2	Advanced breast cancer	NCT01763931
*Ganetespib*	HIF1A/Hsp90	3	Advanced NSCLC	NCT01798485
**b. Inhibiting HIF-targets involved in the regulation of acid TM**				
*SLC-0111*	CAIX	1	Advanced solid tumors	NCT02215850
*DTP348*	CAIX	1	Advanced solid tumors	NCT02216669
**c. Inhibiting HIF-targets involved in the Epithelial Mesenchymal Transition**				
*Rilotumumab*	MET/HGF/SF	3	Advanced gastric cancer	NCT01697072
*Crizotinib* *Axitinib*	MET/ALK	1b	Advanced solid tumors	NCT01999972
*Cabozantinib*	MET/AXL RET/ROS1/NTRK1	2	Advanced NSCLC	NCT01639508
**d. Targeting mitochondrion dysfunction**				
*CPI-613*	Mitochondria	1	Metastatic colorectal cancer	NCT02232152
*Fenofibrate*	Mitochondria	2	Advanced multiple myeloma	NCT01965834
*Antibacterial agents*	Mitochondria	2	Malignant diseases	NCT02366884
***Anti-bacterial pro-drug activated in hypoxia***				
*TH302*	Hypoxic TM	3	Soft Tissue sarcoma	NCT01440088
**e. Targeting stromal-epithelial metabolic coupling**				
*Metformin*	MCT4	0	Head and Neck squamous cells cancer	NCT02083692
*Topotecan/Acetylcysteine*	MCT4/CAV1	2	Advanced ovarian cancer	NCT02569957

## THERAPEUTIC APPROACHES IN ADVANCED CLINICAL TRIALS TARGETING THE ADAPTATION TO HTM

### Targeting HIF1A

#### Digoxin/DIG-HIF

Digoxin, a cardiac glycoside, it has been shown to have anti-cancer activity due to the inhibition of *HIF1A* synthesis, *in vitro* and *in vivo* in several solid tumors [76-78]. Currently, Digoxin is involved in a phase 2 clinical trial (https://clinicaltrials.gov/ct2/show/NCT01763931) as a novel inhibitor of *HIF1A* in breast cancer. It will be administrated to breast cancer patients for 2 weeks prior to surgery. During the two weeks of daily oral digoxin therapy, researchers will measure, by immunohistochemistry, the expression of *HIF1A* protein in surgically resected breast cancer tissues. This clinical trial will be useful also to evaluate adverse events, to assess safety and tolerability of Digoxin in the pre-surgical breast cancer patients according to Common Terminology Criteria for Adverse Events, version 4.

### Ganetespib

The chaperone Hsp90 is involved in tumor growth, angiogenesis and cancer stem cells formation [79, 80]. Its pathway leads to the activation of several oncogenic proteins including HIF1A. It has been shown that Ganetespib (5-[2,4-dihydroxy-5-(1-methylethyl)phenyl]-4-(1-methyl-1H-indol-5-yl)-2,4-dihydro-3H- 1,2,4-triazol-3-one) is able to increase the proteasome-mediated degradation of Hsp90. By this mechanism Ganetespib can inhibit *HIF1A* in a TNBC mouse model [81]. This drug was already administered as mono-therapy in patients with genotypically defined advanced non-small cell lung cancer (NSCLC) in a multicenter phase 2 study and showed manageable side effects and an OS improvement of 11 months (https://www.clinicaltrials.gov/ct2/show/record/NCT01798485) [82]. Currently, Ganetespib is in a phase 3 study in combination with docetaxel in patients with advanced NSCLC (https://www.clinicaltrials.gov/ct2/show/NCT01798485). This clinical trial aims to the identification of a possible synergism between Ganetespib (150 mg/m2) and Docetaxel (75 mg/m2) to propose a more effective anti-cancer therapy than Docetaxel alone.

### Inhibiting HIF-targets involved in the regulation of acid TM

CAIX is a hypoxia-inducible metal-enzyme that promotes cancer cell survival and invasion in HTM via HIF activation [83]. Its role is to catalyze the reversible hydration of carbon dioxide to bicarbonate and protons (H_2_O + CO_2_ to HCO_3_^−^ + H^+^) regulating cellular pH. It is expressed selectively on the cell surface of tumor cells, particularly in the CSCs, [83] and it is one of the major factors contributing to cancer cell survival and metastasis. CAIX is highly expressed in breast cancer and PDAC and it has been shown as biomarker of poor prognosis for metastasis development and survival [83-85]. Further work has established a critical role of CAIX expression in the maintenance of EMT phenotype [84], “stem cell” function and tumor heterogeneity induced by hypoxia [86, 87].

### SLC-0111

SLC-0111, as small molecule, it is able to reach the hypoxic niches and selectively bind and inhibit CAIX. Pre-clinical pharmacokinetic and toxicology analysis of SLC-0111 has been completed, demonstrating little toxicity and positive effects for the treatment of various tumors. Currently, SLC-0111 is involved in a phase I, Multi-center, Open-label, study (https://clinicaltrials.gov/ct2/show/NCT02215850). In this prospective single arm study, SLC-0111 will be given once daily in 28 day cycles, in subjects with advanced solid tumors. The aim is to investigate its safety, tolerability, and pharmacokinetic and to gain some information about its effectiveness in treating cancer.

### DTP348

DTP348, namely 2-(2-methyl-5-nitro-1H-imidazol-1-yl) ethylsulfamide, was designed to target hypoxic tumor regions using low doses. Interestingly, this molecule is able to incorporate, in the same scaffold, different functionalities: the inhibition of CAIX, the acidification of the intracellular pH, and the radio-sensitization in HTM [88]. This oral dual CAIX inhibitor/radio-sensitizer, is currently in a phase I multicenter, open-label, dose-escalation study (https://clinicaltrials.gov/ct2/show/NCT02216669). The clinical trial will investigate the effects of DTP348 alone and in combination with radiotherapy in patients with advanced solid tumors, to determine its recommended phase II Dose, safety and tolerability. Once defined these parameters, DTP348 will be administered every day for 7 weeks to evaluate its pharmacokinetic and anti-cancer activity.

### Inhibiting HIF-targets involved in the Epithelial Mesenchymal Transition

HGF is the natural ligand of *MET* proto-oncogene, a receptor tyrosine kinase [89, 90]. It has been shown that the activation of the HGF/MET pathway induces EMT generating a mesenchymal population more tumorigenic and chemo-resistant than the parental one [91]. HGF/MET are induced by *HIF1A* [92] and it has been shown that *HIF1A* overexpression increases HGF's mRNA stability [93].

### Rilotumumab

Rilotumumab is a human monoclonal antibody immunoglobulin G type 2, that blocks the binding of HGF/SF to its receptor MET. Rilotumumab is currently in a phase 3, multicenter, randomized, double-blind, placebo controlled study, of epirubicin, cisplatin & capecitabine (ECX) or untreated advanced MET-positive gastric or gastroesophageal junction (GEJ) adenocarcinoma (https://clinicaltrials.gov/ct2/show/NCT01697072). The aim of the study is to evaluate if the treatment of ECX in combination with Rilotumumab (15 mg/kg, IV every 21 days) leads to a better clinical outcome, evaluating OS, PFS, time to progression, objective response rate (ORR), disease control rate, time to response and immunogenicity in subjects with un-resettable locally advanced or metastatic *MET* positive gastric or GEJ cancer.

### Crizotinib/axitinib

The hypothesis already discussed in the first part of this review that MET and HGF contribute to VEGF inhibitor resistance, is currently tested in a phase 1b, open label, dose escalation study combining Axitinib (N-methyl-2-[[3-[(E)-2-pyridin-2-ylethenyl]-1H-indazol-6-yl]sulfanyl]benzamide) with Crizotinib (3-[(\1R)-\1-(\2,6-dichloro-3-fluorophenyl)ethoxy]-5-(1-piperidin-4-ylpyrazol-4-yl)pyridin-2-amine) in patients with advanced solid tumors (https://clinicaltrials.gov/ct2/show/NCT01999972). Axitinib is a potent and selective tyrosine kinase inhibitor of VEGF receptor (VEGFR) 1, 2, and 3 [94]. Crizotinib is a *MET* and *ALK* inhibitor, actually in use for patients with *ALK*-positive NSCLC [95]. This clinical trial is really innovative and complex, since this is the first time that Axitinib and Crizotinib are administered in combination. The first part of the study will be essential to evaluate if the oral administration of Axitinib (2-5 mg, twice daily for 28 days cycles) and Crizotinib (200-250 mg, twice daily for 28 days cycles) has an anti-cancer activity in advanced solid tumors. Moreover in this first part the effective dosage that will be administered, will be defined in advanced RCC to evaluate safety, pharmacokinetics and pharmacodynamics.

### Cabozantinib

Cabozantinib (1-N-[4-(6,7-dimethoxyquinolin-4-yl)oxyphenyl]-1-N’-(4-fluorophenyl)cyclopropane-1,1-dicarboxamide) is an oral medicine that inhibits *MET*, *RET*, *ROS1*, *NTRK1*, and *AXL*. The inhibiting activities of Cabozantinib towards MET and AXL is relevant. In fact, researchers found AXL, a member of receptor tyrosine kinase family [96] to increase EMT phenotype, commonly activated in HTM [97]. A key-role of this increased expression is due again by HIF that directly activates *AXL* gene transcription by binding to the hypoxia-response element within the *AXL* proximal promoter [98]. Recently, it has been demonstrated that increased *AXL* expression could contribute to drug resistance in cancer cell lines. In fact, *AXL* down-regulation inhibits cell survival and overcomes drug resistance *in vitro*. *AXL* could activate the downstream signals such as *AKT* and *ERK1* to promote cell survival and growth. Therefore, AXL inhibitors could be effective in abrogating the growth of tumor cells [69]. Thus, through both MET and AXL inhibitions, Cabozantinib was shown to induce cancer shrinkage and cell growth reduction in medullary thyroid and prostate carcinoma. Currently, a phase 2 study is evaluating Cabozantinib in patients with *RET* fusion- (Group A) and those with *ROS1* or *NTRK1* fusions or increased *MET* or *AXL* activity (Group B) in advanced NSCLC (https://clinicaltrials.gov/ct2/show/NCT01639508). During the period of 28 days cycle, 60 mg of Cabozantinib will be administered to patients orally daily, to evaluate positive and negative effects in tumors with genes changes. Moreover, in group A and B ORR, PFS and OS will be evaluated.

## METABOLIC CHANGES AND THERAPEUTIC TARGETS

### Warburg effect, mitochondria changes and cancer stem cells

**Figure 4:**
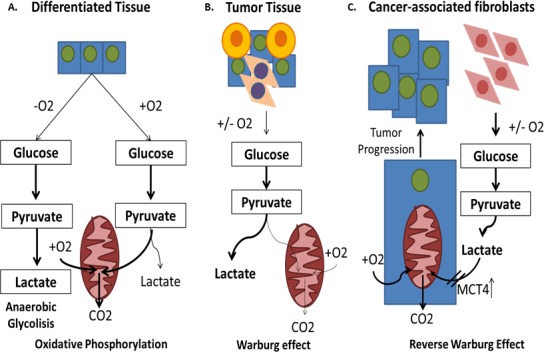
The Warburg effect and the reverse Warburg effect **A.** In normal tissues, glucose is bio-transformed to pyruvate and carried into the mitochondria for the oxidative phosphorylation (OXPHOS). **B.** Most types of cancer engage themselves in glycolysis, irrespective to the presence of oxygen (aerobic glycolysis or Warburg Effect). **C.** Some cancer cells reprogram cancer associated fibroblasts (CAFs) to undergo aerobic glycolysis (WE) and to secrete energy-rich nutrients that feed into mitochondrial oxidative metabolism in cancer cells.

In parallel with the adaptation to a HTM, deep changes are observed also in the mitochondrial functions. A well-known phenomenon commonly found in cancer cells is the so-called Warburg effect (WE). While in normal tissues, glucose is bio-transformed to pyruvate and carried into the mitochondria for the oxidative phosphorylation (OXPHOS) (Figure 4a), in cancer, the glycolysis is 200-fold more active than the normal, either in the presence or absence of oxygen (aerobic glycolysis/WE) leading to high amount of lactate [99] (Figure 4b). Despite the first identification of the WE dates back to 1927, the mechanisms triggering WE are not fully understood, yet. It could be the consequence of damaged mitochondria in cancer cells, or of the adaptation of cancer cells to HTM. The up-regulation of the aerobic glycolysis seems related to oncogene activation. It appears that almost every major oncogene that drives oncogenesis also drives increased glucose metabolism. For example, activating mutations within *BRAF*, *PTEN*, and *KRAS*, were found to have effects in the transcriptional activation and repression of key metabolic enzymes and in the reprogramming of glutamine metabolism [100-102]. WE could be also triggered by the stabilization of *HIF1A*. In fact, once *HIF1A* is stabilized, it is observed that cancer cells increase glycolysis, where a good proportion of glucose is converted into lactate instead going through OXPHOS, therefore creating an acidic microenvironment and leading to an inefficient ATP production [103]. It is well-established that this change is important for survival in hypoxic conditions and it correlates also with CSCs formation. In fact, mitochondria show differences between CSCs and non-CSCs. For example, CSCs from lung cancer and leukemia showed low mitochondrial DNA content, OXPHOS, oxygen consumption rate, intracellular ATP and an enrichment of specific biomarkers (ALDH, CD24, CD44, CD133) responsible for their formation and survival [104]. However, these facts should not mislead regarding to the role of mitochondria in CSCs. In fact, it was also observed that the clonal expansion and survival of CSCs is, actually, dependent on mitochondrial biogenesis suggesting that the mitochondrial metabolism, although reduced, is crucial in CSC survival. In particular, CSCs have been shown to be dependent on OXPHOS. In fact, when an inhibitor of ATP-synthase activity is used, CSCs usually are killed [105]. Although, a reduced mitochondrion activity is observed in cancer, in oncocytoma a paradoxical effect is observed. These “oxyphilic” tumors (typically RCC, ovarian or thyroid cancer) have an impressively abnormal number of mitochondria in their cytoplasm [106-109]. A model was proposed where cancer genes could be responsible for a reduced mitochondria metabolism leading to an inefficient ATP production, and where the mitochondrial hyperplasia is a consequence of a compensatory effect in the attempt, unsuccessful, to restore the normal ATP production [110]. Among the players discovered to be involved in WE and related metabolic changes the Hsp90 chaperon has a role in maintaining energy production under nutrient deprivation [111]. Moreover, SLC2A1 and MCT4 were found frankly over-expressed in cancer cells in correlation to an improved glucose supply, and lactate disposal [112]. In addition, in gastric cancer also *MACC1* was found up-regulated and it was found to contribute in triggering the WE through the up-regulation of glycolytic enzymes [113]. It has been hypothesized that the changes in mitochondrion activity could be a weak-point for CSCs [114]. Thus, novel therapeutic approaches were proposed based on the use of antibiotics targeting mitochondrion [105], in the reminiscence of its prokaryotic origins. Five classes of mitochondrial-targeted antibiotics including erythromycin, tetracycline, glycylcycline, and chloramphenicol effectively reduce CSCs formation [105, 115] and similar results were obtained with oligomycin A, an inhibitor of the mitochondrial ATP synthase [115]. When considering more in general specific mitochondrion inhibitors, a recent work showed that a specific Monocarboxylate Transporters (MCTs) inhibitor (AR-C155858), which blocks the cellular uptake of mitochondrial fuels, such as lactate, could effectively reduce the formation of CSCs [115]. Moreover, novel molecules, such as NV-128, an isoflavone-derivative, showed a significant activity in reducing the mitochondrial function (with decrease in ATP, COX1, and COX4 levels), leading to increased mitochondrial superoxide and hydrogen peroxide. This promoted a state of cellular starvation and oxidation that activated two independent pathways: (i) AMPKa1 pathway leading to MTOR inhibition; and (ii) mitochondrial MAPK/ERK kinase/extracellular signal pathway, leading to loss of mitochondrial membrane potential [116] with an inhibition of CSCs formation. It should be stressed that normal cells appeared more resistant to these inhibitors as compared to CSCs. This fact should provide a wide therapeutic window for reducing the side effects for cancer patients.

### Reverse Warburg Effect and metabolic symbiosis

Once the research on cancer metabolism advances, it was found that WE is not a general rule. In fact, they are highly flexible in adapting to the micro-environmental conditions and they can easily reactivate the mitochondrial OXPHOS [117]. Differences in the metabolic phenotype can even occur within a single tumor mass [118]. Thus, for some tumors, cancer cells are found not completely dependent on accelerated glycolysis. Interestingly, it was discovered that they are “fed” through the activation of normal stromal cells surrounding cancer cells [119]. For example, it has been shown that the activation of *HIF1A* occurs in carcinoma-associated fibroblasts (CAFs) and it enhances aerobic glycolysis and lactate production, which is converted to pyruvate and utilized for mitochondrial OXPHOS in cancer cells [120, 121]. This phenomenon has been called “reverse WE” (RWE), which indicates increased aerobic glycolysis of stromal cells (CAFs) adjacent to cancer cells, which secrete energy-rich nutrients that feed cancer cells [122], leading to an acidic TM, increased tumor growth and malignant behaviors [123]. This host-parasite relationship is characterized also by the loss of caveolin-1 (CAV1), a molecule that has been shown to be responsible for *HIF1A* activation in stromal cells [120] and by the increase of MCTs and in particular MCT4, a transporter responsible for the regulation of both energetic supply and intracellular pH [124] (Figure 4c). Based on this consideration, the development of anticancer agents that target the enzymes involved in glycolysis appears to be promising.

## NOVEL THERAPEUTIC APPROACHES TARGETING METABOLIC CHANGES

### Targeting mitochondrion dysfunction

The mitochondrion changes discussed in the last two sections prompted clinical researchers to focus on the identification of new compounds able to inhibit some of the involved molecules. Hereon, we will describe novel therapeutic agents developed to these purposes (Table 1, Figure 3).

### CPI-613

CPI-613 (6,8-bis(benzylsulfanyl)octanoic acid) is a molecule able to kill cancer cells through the shutdown of mitochondria. Consequently the deprivation of energy and other supplies, lead to the inhibition of cell growth. CPI-613 is currently involved in a pilot phase I trial (https://clinicaltrials.gov/ct2/show/NCT02232152) in combination with fluorouracil in patients with non-resettable metastatic colorectal cancer. The aim of the study is to evaluate if there is a synergism between a chemotherapy drug (fluorouracil, administered IV over 46 hours on days 2-4) and an inhibitor of mitochondria (CP-613, administered IV over 2 hours on days 1-4) to determine the Maximum Tolerated Dose, pharmacokinetic and safety in patients who have failed FOLFOX (leucovorin calcium, fluorouracil and oxaliplatin), or FOLFIRI (leucovorin calcium, fluorouracil, and irinotecan hydrochloride) therapies.

### Fenofibrate

Fenofibrate (propan-2-yl 2-[4-(4-chlorobenzoyl)phenoxy]-2-methylpropanoate), that is currently in use to treat hyperlipoproteinemia (i.e high level of cholesterol and/or triglycerides) [125], has been shown to inhibit mitochondrial function by blocking ATP synthesis and inhibiting electron transport at NADH-ubiquinone (UQ) oxidoreductase. This inhibition leads to the induction of a stress signal known as the unfolded protein response and subsequently apoptosis, in particular in multiple myeloma. Thus fenofibrate is now studied in a phase II clinical trial (https://clinicaltrials.gov/ct2/show/NCT01965834) with the aim to determine response rate, safety and tolerability in multiple myeloma patients.

### Anti-bacterial agents

A combination of two selected antibiotics from doxycycline, paramomycin, clarithromycin, clindamycin, dapsone, miltefosine, targeting mithocondria and with documented anti-cancer properties are currently employed in a phase 2 clinical trial (https://clinicaltrials.gov/ct2/show/study/NCT02366884) alone and/or in combination with anti-fungal and anti-protozoal agents. The aim of this study is to investigate the clinical efficacy of these agents in patients with malignant disease confirmed histologically that is considered untreatable, progressive and fatal within the next 16 months. Tumor regression is expected within 6 months of treatment and to avoid tumor recurrence the treatment will be continued until 10-12 months. This drug repositioning is interesting because we already know safety, tolerability and side effects.

### Anti-bacterial pro-drug activated in hypoxia: TH-302

TH-302 is a prodrug that is activated only under hypoxic conditions; this characteristic prevents the production of a broad systemic toxicity, typical of chemotherapy. Within regions of tumor hypoxia, TH-302 releases a potent DNA alkylating agent called bromo-isophosphoramide mustard [126] that is responsible for blocking the replication and transcription of DNA, leading to cancer cell growth inhibition. Moreover, once TH-302 is activated in hypoxia, it can diffuse to surrounding regions inhibiting the cell growth also in oxygenated cancer tissues, via a “bystander effect” [127]. To date, TH-302 is involved in a randomized phase 3, multicenter, open-label study, in combination with Doxorubicin in patients with locally advanced un-resettable or metastatic soft tissue sarcoma (https://clinicaltrials.gov/ct2/show/NCT01440088). The clinical trial aims to evaluate if Doxorubicin that usually has poor tissue penetration and acts in regions of tumors that are located in proximity to the tumor vessels, in combination with TH-302 could increase the OS in patients with cancers where the presence of hypoxia is high, as shown in soft tissue sarcomas [128]. TH-302 will be administered by IV infusion on day 1 and 8 for 21-days. Doxorubicin will be administered on day 1, after the TH-302 infusion. The pharmacokinetics of each compound will be investigated.

### Targeting stromal-epithelial metabolic coupling (RWE)

#### Metformin

Metformin (1,1-Dimethylbiguanide) is an antidiabetic medication used particularly for the treatment of type 2 diabetes, decreasing hyperglycemia primarily by suppressing gluconeogenesis. Recently metformin is used also for the polycystic ovary syndrome in patients with insulin resistance [129]. Currently metformin is involved in a phase 0 clinical trial (https://clinicaltrials.gov/ct2/show/study/NCT02083692) in patients with head and neck squamous cell cancer. The main purpose of the study is to evaluate if metformin is responsible for a decrease of MCT4 expression in fibroblast and its effect on tumor metabolism.

#### Topotecan/acetylcysteine

Topotecan (9-Dimethylaminomethyl-10-hydroxycamptothecin) is an anticancer agent used in particular for the treatment of ovarian cancer. Acetylcysteine (N-Acetyl-L-cysteine) is used mainly as a mucolytic agent and it seems to block stromal induction of MCT4 [130]. Currently a phase 2 study (https://clinicaltrials.gov/ct2/show/study/NCT02569957) is recruiting patients with ovarian cancer. The aim of this study is to evaluate if the administration of acetylcysteine in combination with topotecan, can inhibit the cancer cell growth, due to changes in *MCT4*, *CAV1* and *HIF1A*.

## CONCLUSIONS

Tumor hypoxia is responsible for radio- and chemo-resistance as well as predisposing for increased CSCs formation and tumor metastases. Pro-angiogenic factors are synthesized and released from tumor, stromal, endothelial, and myeloid cells in response to HTM which are commonly found during cancer progression. The formation of an aberrant and heterogeneous vascular network is a key pathological event in the multistep process of tumor growth and metastasis. The use of VEGF inhibitors, such as avastin, gives some results, especially in colorectal, breast, lung cancer and glioblastoma where it is able to induce vascular normalization and reducing hypoxia (leading to improved efficacy of conventional therapies) [131] nevertheless anti-angiogenic drugs have been shown not to be always effective in the treatment of cancer, particularly for the highly aggressive ones [12]. These considerations have raised the questions that probably angiogenesis is not the main responsible of the tumor progression, shifting the focus on the metabolic changes associated with HTM. It has been thought that inhibiting the response to acidosis and hypoxia and blocking the proteins strictly correlated to HIF1A, EMT and mitochondria functions could be a promising way to treat this disease and worth to be further investigated. The clinical trials described in this review will provide in relatively short time new answers to the researchers of whether this approach will yield results worth to be fully undertaken.
